# Supplementary material for: Objective Biomarkers of Outdoor Activity (Vitamin D and CUVAF) in Young Adults with Myopia During and After the SARS-CoV-2 Pandemic
Source: Biomedicines. 2025 Aug 21;13(8):2042. doi: 10.3390/biomedicines13082042 (PMC12383495; doi:10.3390/biomedicines13082042)
Supplement: Supplementary file 1 [file biomedicines-13-02042-s001.zip › biomedicines-3733007-supplementary.pdf]

biomedicines

MDPI

Supplementary Materials:

Table S1: Habits related to sun exposure and skin protection in young Colombians during and after the SARS-CoV-2 pandemic.

	During the Pandemic			After the Pandemic		
	Myopes	Control	*p*-Value	Myopes	Control	*p*-Value
**Sunscreen Use**						
Yes	8 (44.4)	10 (55.6)		11 (55.0)	9 (45.0)	
No	19 (54.3)	16 (45.7)	0.227*	14 (51.9)	13 (48.1)	0.929
Sometimes	5 (83.3)	1 (16.7)		7 (58.3)	5 (41.7)	
**Occupation Mode**						
In-person	8 (80.0)	2 (20.0)		11 (42.3)	15 (57.7)	
Hybrid	8 (47.1)	9 (52.9)	0.175	10 (47.6)	11 (52.4)	0.013*
Virtual	16 (50.0)	16 (50.0)		11 (91.7)	1 (8.3)	
**Type of illumination**						
Artificial	14 (63.6)	8 (36.4)		11 (52.4)	10 (47.6)	
Solar	1 (25.0)	3 (75.0)	0.315*	2 (40.0)	3 (60.0)	0.746*
Solar and Artificial	17 (51.5)	16 (48.5)		19 (57.6)	14 (42.4)	
**Time (hours)**	(Mean ± SD)			(Mean ± SD)		
Solar exposure	2.06 ± 1.48	2.04 ± 1.48	0.915	2.44 ± 1.62	2.12 ± 1.07	0.535
Device use time	9.81 ± 4.53	7.85 ± 3.79	0.082	9.09 ± 3.61	7.59 ± 3.33	0.175

* Fisher's Exact Test; Mann-Whitney U test for quantitative data.

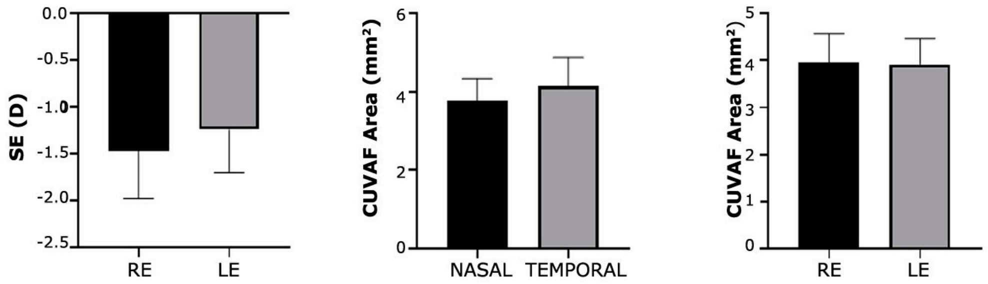

Figure S1: Differences in Spherical equivalents and CUVAF between right and left eyes, and differences in CUVAF between nasal and temporal area of the conjunctiva. No significant differences were found.
